# Chromatin phase separated nanoregions explored by polymer cross-linker models and reconstructed from single particle trajectories

**DOI:** 10.1371/journal.pcbi.1011794

**Published:** 2024-01-24

**Authors:** Andrea Papale, David Holcman

**Affiliations:** 1 Group of Computational Biology and Applied Mathemathics, Ecole Normale Supérieure, IBENS, Université PSL, Paris, France; 2 Churchill College, University of Cambridge, United Kingdom; Massachusetts Institute of Technology, UNITED STATES

## Abstract

Phase separated domains (PSDs) are ubiquitous in cell biology, representing nanoregions of high molecular concentration. PSDs appear at diverse cellular domains, such as neuronal synapses but also in eukaryotic cell nucleus, limiting the access of transcription factors and thus preventing gene expression. We develop a generalized cross-linker polymer model, to study PSDs: we show that increasing the number of cross-linkers induces a polymer condensation, preventing access of diffusing molecules. To investigate how the PSDs restrict the motion of diffusing molecules, we compute the mean residence and first escaping times. Finally, we develop a method based on mean-square-displacement of single particle trajectories to reconstruct the properties of PSDs from the continuum range of anomalous exponents. We also show here that PSD generated by polymers do not induces a long-range attracting field (potential well), in contrast with nanodomains at neuronal synapses. To conclude, PSDs can result from condensed chromatin organization, where the number of cross-linkers controls molecular access.

## Author summary

Within the realm of cell biology, phase-separated domains (PSDs) emerge as pervasive nanoregions characterized by high molecular concentrations. These domains manifest in diverse cellular contexts, ranging from neuronal synapses to the nucleus of eukaryotic cells, where they intricately regulate the accessibility of molecules, particularly transcription factors, thereby modulating gene expression. In this study, we present a comprehensive investigation of PSDs through the lens of a generalized cross-linker polymer model. Our model elucidates that an augmentation in the number of cross-linkers initiates polymer condensation, creating a condensed environment that impedes the diffusion of molecules. To unravel the intricate impact of PSDs on molecular motion, we calculate mean residence and first escaping times. Introducing a novel methodology based on the mean-square-displacement of single particle trajectories, we reconstruct PSD properties across a spectrum of anomalous exponents, providing nuanced insights into their dynamic behavior. In contrast to nanodomains at neuronal synapses, PSDs generated by polymers do not create a long-range attractive field, indicating a distinctive organizational principle. In conclusion, this research advances our understanding of PSDs, portraying them as outcomes of condensed chromatin organization, where the quantitative presence of cross-linkers emerges as a pivotal determinant regulating molecular access. These insights contribute to a refined comprehension of molecular dynamics, offering a foundation for further investigations into the functional implications of PSDs in cellular processes.

## 1 Introduction

Chromatin in the cell nucleus is organized uniformly (euchromatin), forming regions associated with gene expression, or in dense heterogeneous regions called heterochromatin, where genes are hardly expressed [1]. Heterochromatin is less accessible to transcription factors [2], remodelers or polymerase. However, the formation and maintenance of heterochromatin microdomains remain unclear, although remodelers such as histone HP1, NURD remodelers or transcription factors can bind chromatin to form local foci through specific interactions [3–8] and can also modify the local condensation. Foci can also be generated during double-stranded DNA break [9, 10], the property of which can be revealed by single particle trajectories (SPTs). In the case of tagged NURD remodeler, SPTs reveal chromatin organization, where decondensation is associated with an increase of the anomalous exponent [11–13], a parameter that quantifies how the mean square displacement depend on the time increment. This decondensation is associated to increase of the confinement length, that characterizes the confined volume (in 3d) or the surface (in 2d) visited by trajectories.

Phase Separated Domains (PSDs) [14–16] are regions with a size ranging from hundreds nanometers to microns, that can be found in cell biology ranging from neuronal organizations [17], post-synaptic density, synaptic organization [17, 18], immune synapses or nucleus organization, possibly originated from disorder aggregates [19–21], or local chromatin interaction [22–25]. We recall that a PSD is defined in physical terms as a condensate, which refers to membraneless, dynamical, and spatially organized assemblies of biomolecules within cells. These condensates are formed through a process called phase separation, which could be driven by weak, multivalent interactions among molecules such as proteins and nucleic acids. The interactions lead to the separation of these biomolecules from the surrounding cellular environment, creating distinct compartments or condensates. The goal of this manuscript is not to explore all possible mechanisms involved in condensate formation observed in cell biology but to characterize how increasing the number of cross-linkers can lead to a region like structure that can isolate a polymer cross-linked ensemble, preventing inward or outward fluxes of diffusing molecules. Motions in PSDs is often characterized by a large-range of transient to permanent trappings, that can be characterized by potential wells [21]. Chromatin is also organized in large regions called Topological Associated Domains (TADs), regions with enhanced local interactions, revealed by population analysis of Hi-C maps at Mbps scale. TADs results from an enriched sub-contact interaction reveal by an increased contact probability in a submatrix obtained from population Hi-C averaging. It appears as a block sub-matrix in the contact map matrix. Although it is difficult for two TADs to interpenetrate, freely moving molecules should be able to penetrate a single TADs. We will explore here how adding connector to TADs could lead to a transition to PSDs.

It remains unclear how PSDs affect the dynamics of stochastic particles and how the exchange rate is controlled across. Chromatin regions contain a diversity of structures at multiple scales; these structures include A/B compartments [3], TADs, nucleolus, lamina and liquid-like structures [26]. PSDs are precisely supposed to be isolated from the rest of the nucleus. However, we will explore here how proteins could still diffuse outside by possible small transient funnels.

We explore here how PSDs can be generated and regulate the in and outflux of diffusing molecules. Several polymer models have been used to investigate the spatial organization of chromatin [27] at various scales, including TADs, based on diffusive binders with specific binding sites [28–30], attractive or heterogeneous interactions among epigenomic domains [25, 31–33] or random cross-linkers [6, 34–37]. Using cross-link polymer models, we explore how local high density chromatin regions can emerge and form PSD. To quantify the ability to prevent molecular exchange, we explore how diffusing molecules can be excluded from PSDs due to spatial constraint and volume exclusion. By increasing the number of cross-linkers, PSDs emerge and the reduced volume inside the condensed chromatin can prevent most diffusing molecules from accessing. We characterize the PSDs by estimating a penetration length across their fuzzy boundary. To quantify the porosity of the PSD boundary to Brownian molecules, we compute the mean residence time and the first escaping times [38, 39]. The deviation from diffusion due to chromatin organization is revealed by the spectrum of anomalous exponent computed over SPTs, that decays from the center to the periphery and also by increasing the number of connectors.

## 2 Results

### 2.1 Modeling chromatin phase separation with a Random-cross-link Polymer model

**Fig 1:**
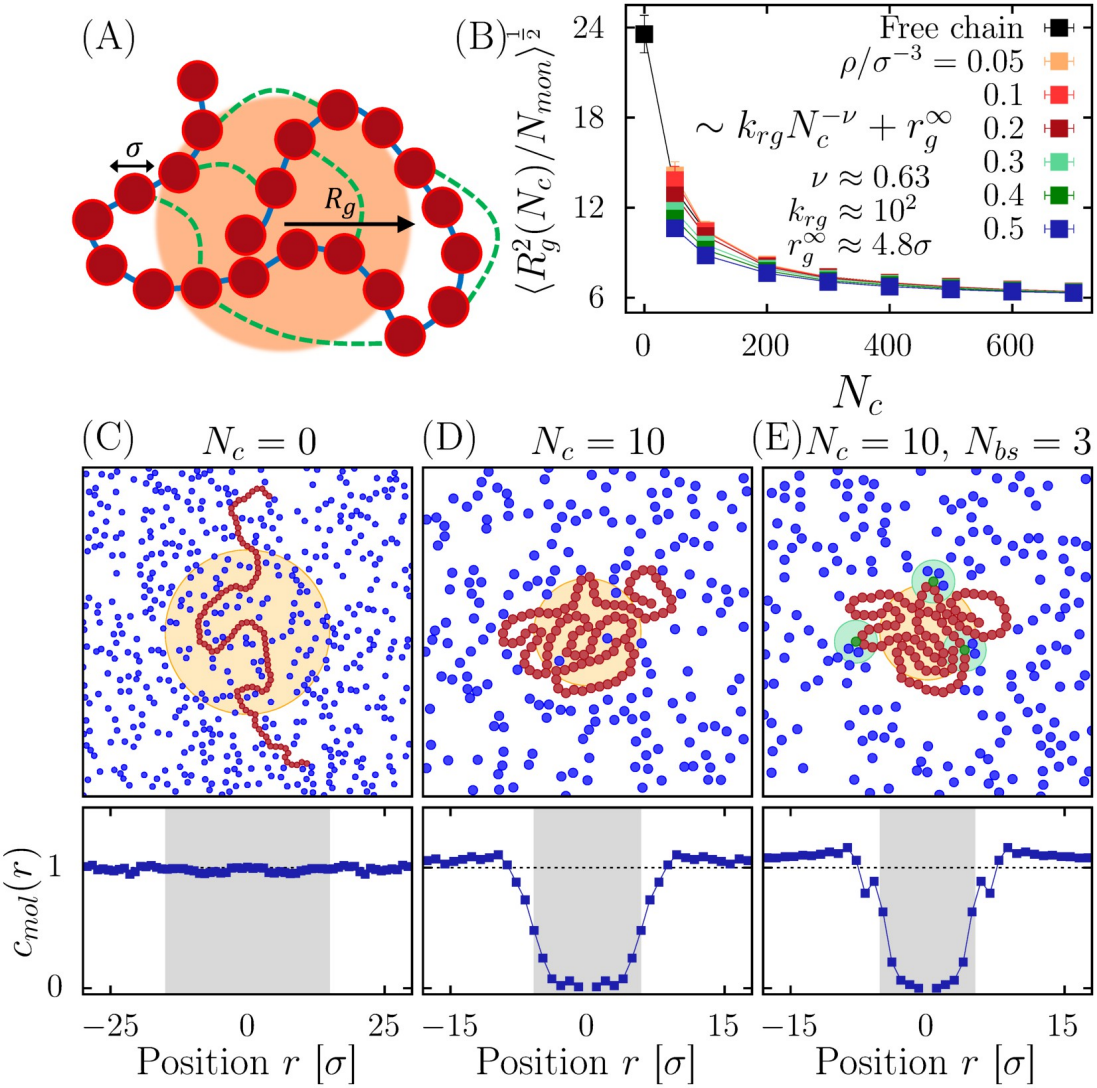
**A**. Scheme of local chromatin reconstruction based on a cross-linked polymer model (red bead of diameter *σ*) connected by springs (blue) with random connectors (green dots). The ball *B*(*R*_*g*_) (orange domain) defines the radius gyration. **B**. Mean gyration radius vs number of random cross-linkers *N*_*c*_, for various densities *ρ*: a smooth transition occurs from a swollen chain to a compact state (*N*_*mon*_ = 2000). **C-E**. Linear chain (red monomers) without random connectors embedded in *N*_*mol*_ Brownian molecules (blue). Random connectors drive the free particles outside *B*(*R*_*g*_). When there are *N*_*bs*_ binding sites, the concentration of molecules *c*_*mol*_(*r*) at distance *r* from the center, is depleted in *B*(*R*_*g*_) (lower panels).

To investigate how chromatin condensation can generate phase-separated domains, we generalize the random cross-linker (RCL) model [34, 40, 41], which consists of a Rouse polymer with randomly added cross-linkers, but fixed for a given configuration. Although cross-linkers, such as HP1, cohesin, and condensin, are dynamically moving with stochastic binding/unbinding and diffusive or active movement along the chromatin chain, we do not account here for these dynamical aspects, as we model the steady-state organization of PSD. Indeed, PSDs are stable structure for a much longer time than the tens to hundreds of seconds required for loop formation by these cross-linkers. As we shall see, the exact location of cross-linking binding events, as long as we account for the overall number of bound, should not affect the statistical properties of the PSD at steady-state. Note that the present model is not sufficient to analyze chromatin loop formation. We adopted a coarse-grained semi-flexible chain with volume-excluded interactions modeled by Lennard-Jones forces, following the Kremer-Grest bead-spring polymer model [42, 43]). Each of the *N*_*mon*_ monomers represents a segment of 3 kbps with a size of *σ* = 30*nm*, and additional cross-linkers are chosen at random positions as in the RCL-polymer model [34, 40]. Similarly, we consider that diffusing molecules have a similar size of 3 kb. This scale has been largely considered for several polymer models [42, 43]. A cross-linker consists of a harmonic spring between two randomly chosen monomers (Fig 1A). The chromatin network resulting from *N*_*c*_ random connectors defines a realization and accounts for the local organization induced by cohesin, condensin or CTCF and thereby combination [35, 44–46].

We first investigate the effects of increasing the number of random cross-linkers on an isolated chain revealing a transition from a coil configuration to a globular state, as characterized by the gyration radius 〈*R*_*g*_〉 (Fig 1B, black curve) [40, 47] where 〈.〉 represents the average over simulations and cross-linkers realizations. We found that gyration radius is well approximated by a power-law $\langle R_{g}\rangle ∼k_{rg}N_{c}^{-\nu }+r_{g}^{\infty }$, where *k*_*rg*_ = 10^2^ ± 20 *σ*, *ν* = 0.63 ± 0.05, $r_{g}^{\infty }=4.8\pm 0.3$ *σ*. In the limit of large amount of connectors, *N*_*c*_ → ∞, 〈*R*_*g*_〉 converges to a non-zero constant value $r_{g}^{\infty }$ due to the volume-excluding interactions.

To investigate how the chromatin structure can influence the dynamics and the distribution of random moving molecules, we simulated a RCL-chain with *N*_*mon*_ = 2000 monomers and *N*_*c*_ = {50, 100, ‥, 700} random connectors embedded in a volume containing *N*_*mol*_ = 8000 diffusing molecules of size *σ* that interact with the chromatin via Lennard-Jones volume exclusion forces, Fig 1C and 1D.

We also introduce specific attractive interactions between diffusing molecules and a set of *N*_*bs*_ = {0, 10} selected monomers of the chain (Fig 1E). We performed fixed-volume molecular dynamics simulations [48] in a fixed cubic volume *V* with periodic boundary conditions and the overall density is defined by *ρ* = (*N*_*mon*_ + *N*_*mol*_)/*V* and *ρ*/*σ*^3^ ∈ [0.05, 0.5].

We report that the average gyration radius 〈*R*_*g*_〉 is slightly affected by the presence of the diffusing molecules (Fig 1B), in particular for small *N*_*c*_, the effective density of the polymer increases. The nano-region occupied by the polymer varies dynamically with the chain motion thus we define the boundary of the separated phase domain as the convex ball Ω = *conv*({**r**|**r** − **r**_**CM**_| ≤ 〈**R**_**g**_〉, }, where **r**_**CM**_ is the polymer center of mass and the radius is 〈*R*_*g*_〉. Interestingly, diffusing particles can be excluded from the region Ω as the number of cross-linkers is increasing (Fig 1C and 1D below) even with binding domains (Fig 1E below).

### 2.2 Statistics distribution of diffusive molecules with in a PSD

To study the distribution of Brownian molecules with respect to the PSD, we use as a reference the radial distribution of molecules with respect to the center of mass *CM*

$$\begin{array}{c} g_{\text{mol}}\left(r\right)=\frac{V}{4\pi r^{2}N_{mol}}\langle \sum_{i=1}^{N_{mol}}\delta (r-|{\text{r}}_{\text{i}}-{\text{r}}_{\text{CM}}\left|\right)\rangle . \end{array}$$

Similarly, the distribution of monomers is characterized by

$$\begin{array}{c} g_{\text{mon}}\left(r\right)=\frac{V}{4\pi r^{2}N_{mon}}\langle \sum_{i=1}^{N_{mon}}\delta (r-|{\text{r}}_{\text{i}}-{\text{r}}_{\text{cm}}\left|\right)\rangle \end{array}$$

and the pair correlation function molecules-monomers is given by

$$\begin{array}{c} g_{\text{mol},\text{mon}}\left(r\right)=\frac{V}{4\pi r^{2}N_{mon}N_{mol}}\langle \sum_{i=1}^{N_{mon}}\sum_{j=1}^{N_{mol}}\delta (r-|{\text{r}}_{\text{i}}-{\text{r}}_{\text{j}}\left|\right)\rangle . \end{array}$$

**Fig 2:**
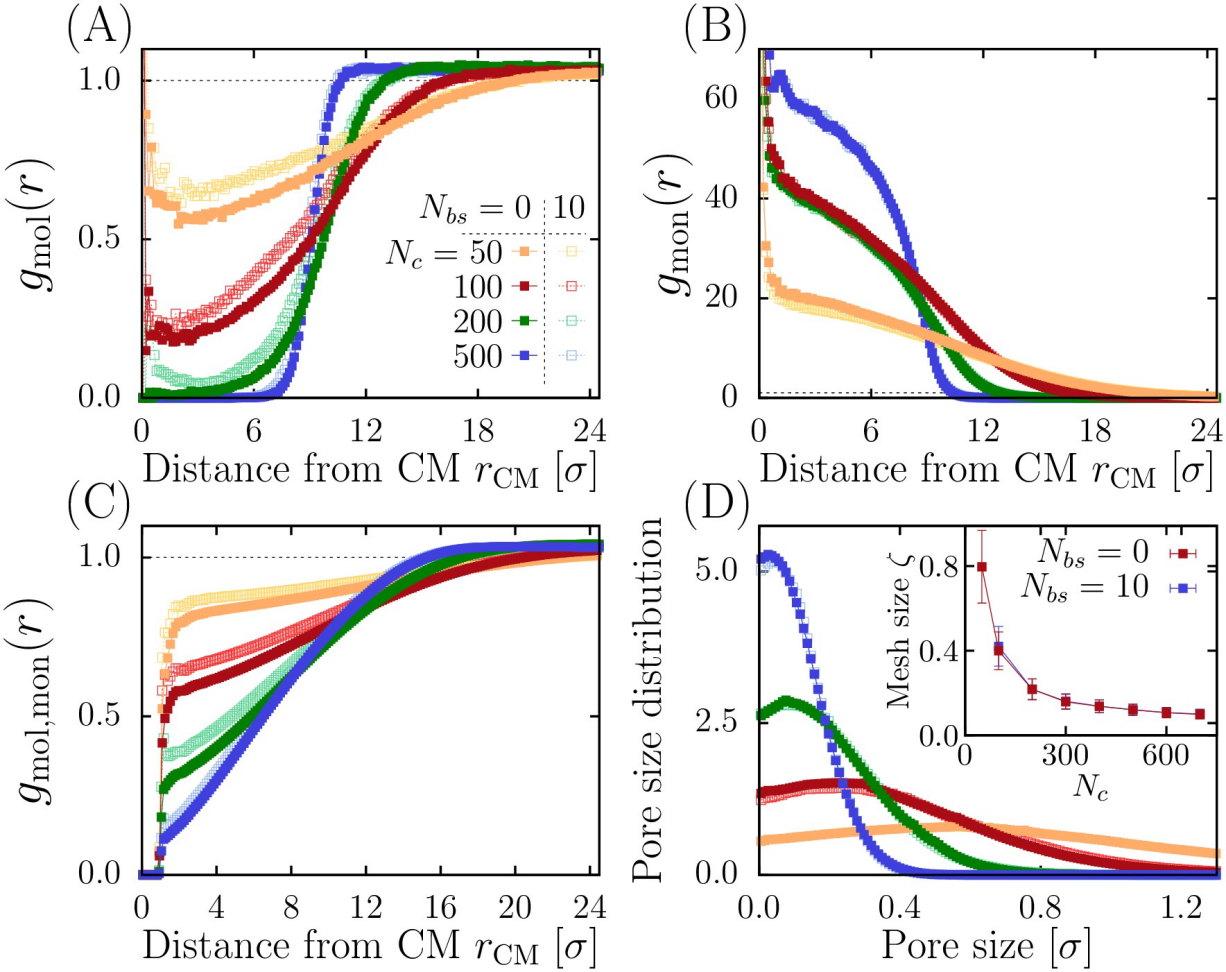
**A**. Molecular radial distribution function *g*_mol_(*r*) for various *N*_*c*_ ∈ {50, 100, 200, 500} at density *ρ* = 0.05*σ*^−3^, compared to the refence constant dashed line. Full (resp. empty) symbols indicate cases with *N*_*bs*_ = 0 (*N*_*bs*_ = 10). **B**. Polymer radial distribution function *g*_mon_(*r*). **C**. Molecules-monomers pair correlation function *g*_mol_(*r*). **D**. Pore size distribution. Inset: average mesh size *ζ* vs. *N*_*c*_.

The radial distribution functions of monomers and molecules reveal that the RCL-chain separates diffusing molecules, a phenomena that is amplified by increasing the number of random connectors (Fig 2A and 2B), regardless of the overall density (see also S1 Fig for the radial pair distribution functions for various density *ρ*). We thus conclude that the presence of random connectors can create a separation between a condensed polymer and interacting molecules.

To further characterize the spatial organization of the RCL-chain, we analyze the available space for diffusion in the region Ω [49, 50] by estimating the pore size distribution *P*_*s*_ from the maximum volume that do not contain any other monomer inside the region (Fig 2D). The mesh size is defined as the mean pore radius *ζ* = 〈*s*〉 = ∫ *sP*_*s*_*ds* that can be approximated as $\zeta ∼k_{\zeta }N_{c}^{-\gamma }+\zeta^{\infty }$. For *N*_*bs*_ = 0 (resp. *N*_*bs*_ = 10) fitting the simulations reveals an exponent *γ* = 1.13 ± 0.01 (1.23 ± 0.04), *k*_*ζ*_ = 60 ± 3 *σ* (100 ± 20 *σ*) and *ζ*^∞^ = 0.062 ± 0.002 *σ* (0.071 ± 0.004 *σ*) (Fig 2D inset). To conclude, increasing the connectors *N*_*c*_ forces the polymer to condense and to progressively exclude random particles, sharpening the boundary of the PSD.

### 2.3 Quantifying the PDS insulation using first passage time analysis

**Fig 3:**
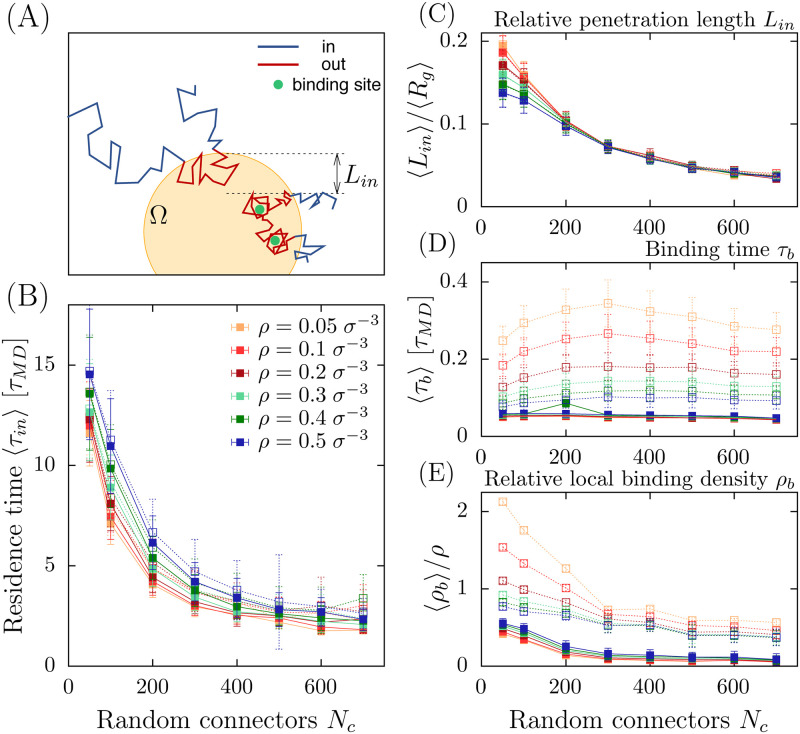
**A**. Schematic representation of molecular trajectories penetrating the phase separeted region Ω over a characteristic length *L*_*in*_ with and without binding sites (green). **B**. Mean time 〈*τ*_*in*_〉 spent by a molecule inside the PSD versus number of connectors *N*_*c*_ for various densities *ρ*. Full (resp. empty) symbols indicate cases with *N*_*bs*_ = 0 (*N*_*bs*_ = 10). **C**. Ratio of the penetration length 〈*L*_*in*_〉 to the gyration radius 〈*R*_*g*_〉 versus *N*_*c*_. **D**. Mean binding time 〈*τ*_*b*_〉 vs *N*_*c*_. **E**. Ratio of the local density *ρ*_*b*_ estimated around the binding sites to the overall density *ρ* (no binding sites).

To further characterize how a PSD is isolated to ambient trafficking molecules, we explore how it can prevent random molecules to penetrate or escape the domain Ω, defined by the condensed chromatin polymer. To estimate the resident time *τ*_*in*_ spent by Brownian molecules inside the nanoregion after crossing its boundary (Fig 3A), we run various simulations and we show this time depends weakly on the overall density of these particles or on the presence of binding sites (Fig 3B).

To further explore the ability of the PSD to prevent molecules from penetrating deeply inside, we defined and then estimated the penetration length *L*_*in*_ of a trajectory before as the maximum length it can go inside the PSD before returning back to the boundary ∂Ω. We find (Fig 3C) that on average particles cannot penetrate more than 15–20% inside even with few connectors. Furthermore, the penetration length *L*_*in*_ decays uniformly with *N*_*c*_.

To investigate the effects of binding sites on the retention time inside Ω, we computed the average binding time *τ*_*b*_ of the Brownian molecules inside the region Ω and found that this time is slightly affected by the number of random connectors (Fig 3D). This result suggests an enhanced turnover of bounded particles which depends on the overall density. Finally, random connectors are sufficient to compact the polymer, leading to a partial shield of the binding sites, thus reducing the number of multiple bonds, as revealed by the local density *ρ*_*b*_ of Brownian particles around the binding sites (Fig 3E).

### 2.4 Mean escape time to quantify PSD insulation

**Fig 4:**
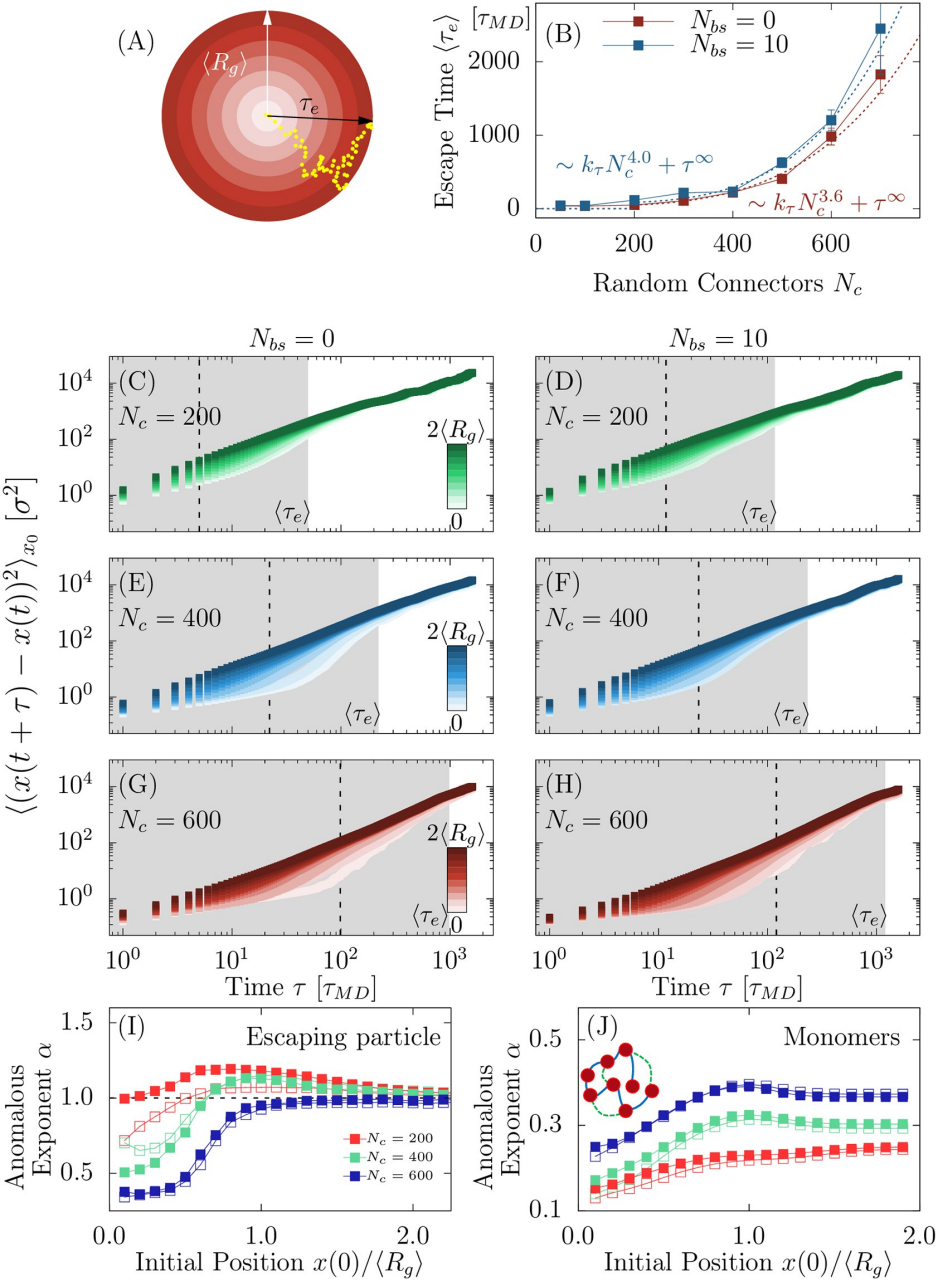
**A**. Schematic representation of a trajectory (yellow) inside the PSD, with the polymer center of mass CM (color shadows). A molecule spends a random time *τ*_*e*_ before crossing the boundary. **B**. Average escaping time 〈*τ*_*e*_〉 from the PSD versus *N*_*c*_ with and without binding sites. **C-H**. MSD of molecules escaping from the PSD for different values *N*_*c*_ = 200, 400, 600, with *N*_*bs*_ = 0 (left column) and *N*_*bs*_ = 10 (right). Curves are colored according the range of the initial position (white inside, dark outside the PSD). Gray regions indicate the mean escape time 〈*τ*_*e*_〉 timescale. The binning length is $\delta x=\frac{1}{10}\langle R_{g}\rangle$. **I**. Anomalous *α*-exponent computed from the MSD of escaping particles in the time interval *τ* ∈ [1, 10^−1^*τ*_*e*_] with respect to the initial radial position *r*. Full (reps. empty) points correspond to *N*_*bs*_ = 0 (resp. *N*_*bs*_ = 10). **J**. Anomalous *α*-exponent computed from the MSD of monomers in the polymer center of mass reference, in the time interval *τ* ∈ [1, 10^−1^*τ*_*e*_] with respect to the initial radial position *r*.

Although PSDs can be isolated from the rest of their local environment, few trajectories could still escape or enter. To investigate their statistical properties, we study how single diffusing molecules positioned at the center of mass *CM* can escape. We run simulations to estimate the mean escape time 〈*τ*_*e*_〉 (Fig 4B) and we found a scaling law $\langle \tau_{e}\rangle ∼k_{\tau }N_{c}^{\eta }+\tau^{0}$, with *η* = 3.6 ± 0.3, *k*_*τ*_ = 4 ⋅ 10^−8^ ± 10^−8^ *τ*_*MD*_, *τ*^0^ = 35 ± 2 *τ*_*MD*_ (no binding) and *η* = 4.0 ± 0.3, *k*_*τ*_ = 2 ⋅ 10^−10^ ± 10^−10^ *τ*_*MD*_, *τ*^0^ = 80 ± 8 *τ*_*MD*_ (with binding). The mean time *τ*^0^ is associated with the diffusing particles escaping the PSD in the absence of connectors (Fig 4A and 4B).

**Fig 5:**
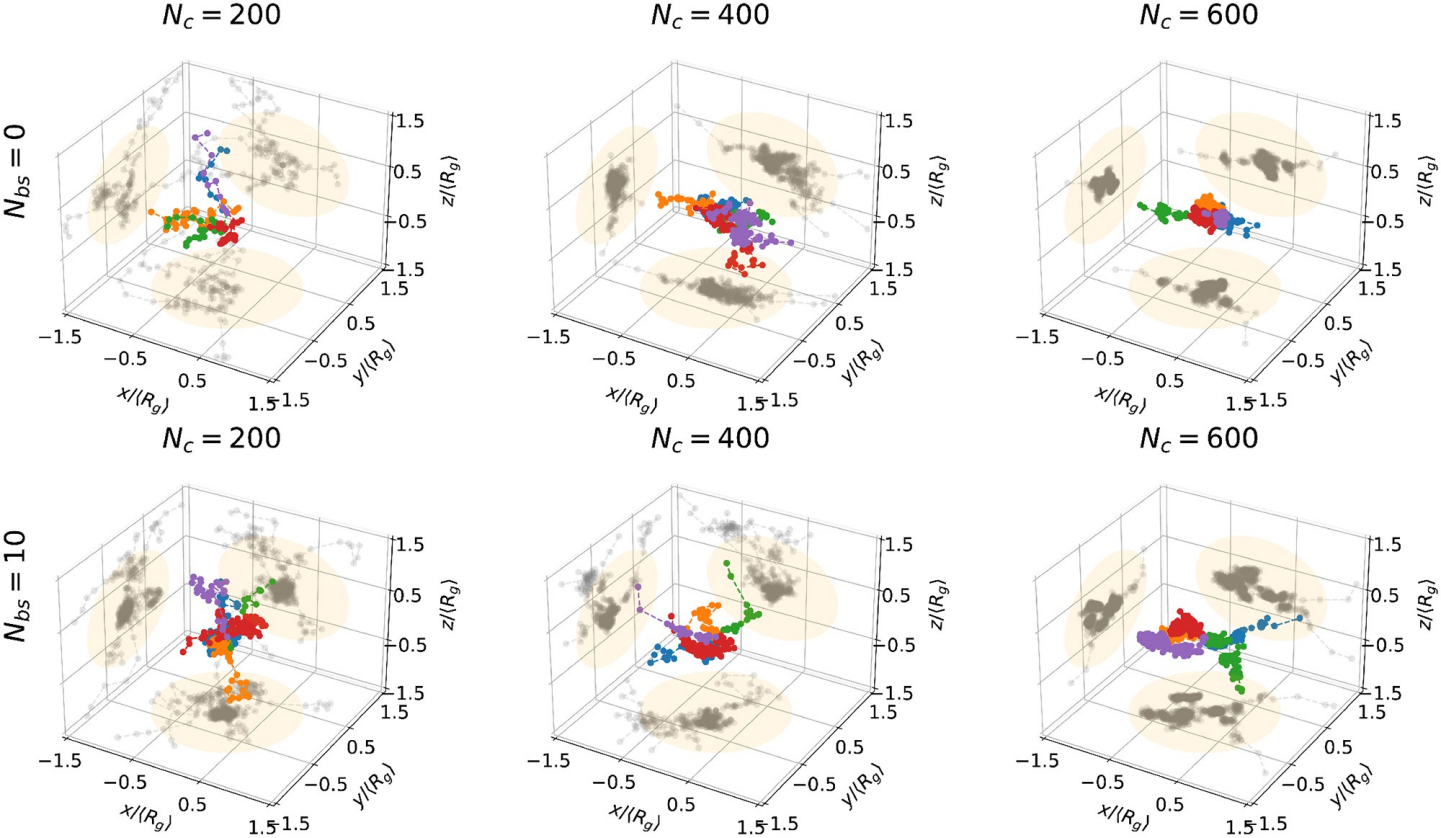
Few examples of trajectories of escaping particles for systems with *N*_*c*_ = 200, 400, 600 (columns) and *N*_*bs*_ = 0, 10 (rows) highlighted with different colors. On each surface the projected trajectories are shown in gray, orange circles represent the projections of the Ω regions defined by the gyration radius.

To study the impact of chromatin condensation on diffusing particles, we analyzed trajectories for various distances |*x*_0_| = *r* (see Fig 5 for trajectory examples) from the polymer CM and computed the average mean square displacement (MSD):

$$\begin{array}{ccc} \left\langle (x(t+\tau )-x(t){)}^{2}|x\left(t\right)\in A_{r}\right\rangle =\frac{1}{N_{run}}\sum_{i=1}^{N_{run}}\frac{1}{N(x_{i}\left(t\right))}\sum_{\left\{i|x_{i}\left(t\right)\in A_{r}\right\}} & & (x_{i}\left(t+\tau \right)-x_{i}\left(t\right){)}^{2}, \end{array}$$

where *i* is the index of a trajectory, *x*(*t*) is the position of the trajectory inside the annulus *A*_*r*_ = (*r*, *r* + *δr*) and the conditional average 〈.|*x*(*t*) ∈ *A*_*r*_〉 is obtained from all initial positions starting in *A*_*r*_ at time *t*. We performed *N*_*run*_ = 100 simulations repeated for *N*_*r*_ = 100 polymer realizations for 2 ⋅ 10^3^ *τ*_*MD*_. By increasing the random connectors, a diffusing molecule trapped inside the PSD remains blocked due the many polymer loops that occupy the available space. An escape route for the diffusing particle (Fig 4B) can however emerge as a rare event, where polymer loops create a transient opening.

To characterize how the polymer organization creating long-range interactions can affect the dynamics of Brownian particles, we computed the MSD functions (Fig 4C–4H), showing a continuous spectrum that depends on the distance *r* from the CM and the number of connectors *N*_*c*_. The MSD of trajectories starting near CM (brighter curve in Fig 4C–4H) shows multiple dynamics, compared to the one starting outside (darker colors). Fitting the MSD curves with ∼ *D*_∞_*τ*^*α*^, we computed the anomalous exponents *α* for escaping molecules and also for monomers where the reference is CM. We find similar behaviors characterized by two regimes: (i) anomalous diffusion where the escaping molecules are progressively squeezed out by the polymer and (ii) normal diffusion when approaching the boundary of the PSD (see comparison in Fig 4I and 4J).

### 2.5 Mechanism to retain diffusing molecules in a phase separated domain is not an attractor

To investigate whether the PSD can retain stochastic particles with the characteristic of a potential well, we assumed that trajectories could result from a coarser spatio-temporal motion following the stochastic process [51, 52]

$$\begin{array}{c} \dot{X}=a\left(X\right)+\sqrt{2}B\left(X\right)\dot{W}, \end{array}$$ (1)

where *a*(***X***) is the drift field and *B*(***X***) is a matrix and $\dot{W}$ is a random noise. The drift in Eq 1 can be recovered from SPTs acquired at any infinitesimal time step Δ*t* by estimating the conditional moments of the trajectory displacements Δ***X*** = ***X***(*t* + Δ*t*) − ***X***(*t*) [52–55]

$$a(x)=\underset{\Delta t\to 0}{\text{lim}}\frac{E[\Delta X(t)\;|\;X(t)=x]}{\Delta t},$$ (2)

The notation E[⋅|X(t)=x] represents averaging over all trajectories that are passing at point *x* at time *t*. To estimate the local drift *a*(***X***) at each point ***X*** and at a fixed time resolution Δ*t*, we use a procedure based on a square grid. The local estimators to recover the vector field consist in grouping points of trajectories within a lattice of square bins *S*(*x*_*k*_, Δ*x*) centered at *x*_*k*_ and of width Δ*x*. For an ensemble of *N* three-dimensional trajectories $\left\{X_{i}(t_{j})=(x_{i}^{\left(1\right)}(t_{j}),x_{i}^{\left(2\right)}(t_{j})),x_{i}^{\left(3\right)}(t_{j})i=1‥N,j=1‥M_{i}\right\}$ with *M*_*i*_ the number of points in trajectory ***X***_*i*_ and successive points recorded with an acquisition time *t*_*j*+1_ − *t*_*j*_ = Δ*t*. The discretization of Eq 2 for the drift *a*(*x*_*k*_) = (*a*^(1)^(*x*_*k*_), *a*^(2)^(*x*_*k*_), *a*^(3)^(*x*_*k*_)) in a bin centered at position *x*_*k*_ is

$$\begin{array}{ccc} a^{\left(u\right)}\left(x_{k}\right) & \approx & \frac{1}{N_{k}}\sum_{i=1}^{N}\sum_{j=0,x_{i}\left(t_{j}\right)\in S(x_{k},\Delta x)}^{M_{i}-1}(\frac{x_{i}^{\left(u\right)}\left(t_{j+1}\right)-x_{i}^{\left(u\right)}\left(t_{j}\right)}{\Delta t}), \end{array}$$ (3)

where *u* = 1..3 and *N*_*k*_ is the number of points *x*_*i*_(*t*_*j*_) falling in the square *S*(*x*_*k*_, *r*).

At this stage, we would like to compare the empirical drift obtained from the trajectories of diffusing particles with the one generated by a parabolic well. We consider the basin of attraction of a truncated elliptic parabola with the associated energy function

$$\begin{array}{c} U\left(X\right)=\{\begin{array}{cc} A[(\frac{x^{\left(1\right)}-\mu^{\left(1\right)}}{a}{)}^{2}+(\frac{x^{\left(2\right)}-\mu^{\left(2\right)}}{b}{)}^{2}+(\frac{x^{\left(3\right)}-\mu^{\left(3\right)}}{c}{)}^{2}-1], & X\in B\\ 0 & \text{otherwise} \end{array} \end{array}$$ (4)

where *A* > 0 and ***X*** = [*x*^(1)^, *x*^(2)^, *x*^(3)^], ***μ*** = [***μ***^(1)^, ***μ***^(2)^, ***μ***^(3)^] is the center of the well, *a*, *b*, *c* are the elliptic semi-axes lengths and the elliptic boundary is defined by

$$\begin{array}{c} B=\{X\text{such}\;\text{that}\;A[(\frac{x^{\left(1\right)}-\mu^{\left(1\right)}}{a}{)}^{2}+(\frac{x^{\left(2\right)}-\mu^{\left(2\right)}}{b}{)}^{2}+(\frac{x^{\left(3\right)}-\mu^{\left(3\right)}}{c}{)}^{2}-1]=0\}. \end{array}$$ (5)

The PSD is centered at ***μ***^(1)^ = ***μ***^(2)^ = ***μ***^(3)^ = 0 and the elliptic semi-axes lengths are approximated by the radius gyration *R*_*g*_. To estimate the attraction coefficient *A*, we use the least-square regression formula

$$\begin{array}{c} A=R_{g}^{2}\frac{1}{2}\frac{\sum_{k=1‥3,i=1}^{M}a^{\left(k\right)}\left(X_{i}\right)x_{i}^{\left(k\right)}}{\sum_{k=1}^{3}\sum_{i=1}^{M}{\left(x_{i}^{\left(k\right)}\right)}^{2}}, \end{array}$$ (6)

where $X_{i}=[x_{i}^{\left(1\right)},x_{i}^{\left(2\right)},x_{i}^{\left(3\right)}]$ (*i* = 1 … *M*) are the centers of the *M* bins.

Finally, we can estimate the quality of the well (parabolic index) based on the residual least square error:

$$\begin{array}{c} S=1-\frac{1}{2}\frac{(\sum_{k=1‥3,i=1}^{M}a^{\left(k\right)}(X_{i})x_{i}^{\left(k\right)}{)}^{2}}{\left(\sum_{k=1}^{3}\sum_{i=1}^{M}{\left(x_{i}^{\left(k\right)}\right)}^{2})(\sum_{i=1}^{M}||a(X_{i}{)||}^{2}\right)}. \end{array}$$ (7)

**Fig 6:**
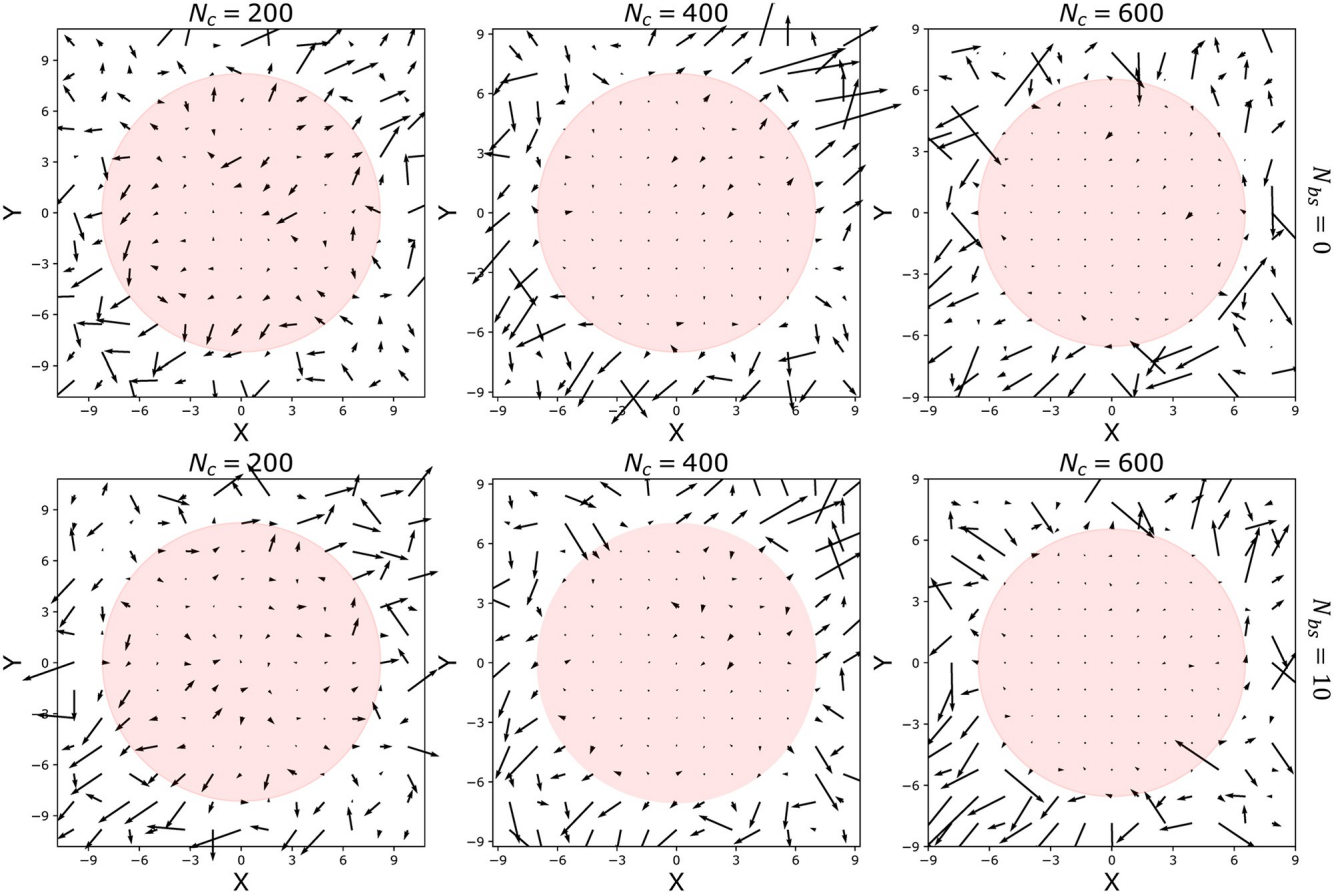
Vector fields on the plane *x*−*y*, *z* = 0, computed from escaping particle trajectories with *N*_*c*_ = 200, 400, 600 connectors (columns) and *N*_*bs*_ = 0, 10 (rows). Circles represent the projections of the Ω regions defined by the gyration radius.

The index *S* ∈ [0, 1] is defined such that *S* → 0 for a drift field generated by a parabolic potential well and *S* → 1 for a random drift vector field, as observed for diffusive motion [51]. When we apply the procedure describe above to recover and characterise a possible drift field inside the PSD. We found that there was no drift associated with the PSD, as summarized in Table 1 below. Thus the escape from MSD is not driven by any drift as shown in Fig 6. The score parameter *S* ≈ 1 for the different parameter values is reported in Table 1. These results show that the PSD (Fig 6) traps stochastic particles with a mechanism different from an attracting potential well.

**Table 1:** A values computed from the simulated trajectories described in Fig 6.

Number of connectors *N*_*c*_	Numbers of binding sites *N*_*bs*_ = 0	*N*_*bs*_ = 10
200	*A* = 2 ⋅ 10^−4^	*A* = 6 ⋅ 10^−5^
400	*A* = 10^−4^	*A* = 9 ⋅ 10^−5^
600	*A* = 5 ⋅ 10^−5^	*A* = 10^−4^

### 2.6 Scaling law for the mean escape time from a PSD

Finally, to investigate how the mean escape time for a stochastic molecule depends on the number of connectors, we use the narrow escape theory [56] allowing us to replace the moving RCL-chain that generates transient obstacle barriers by a partial reflecting boundary at the escape windows. Indeed, as suggested by the escape time results of Fig 4A and 4B, only a small fraction of the boundary is accessible for escape. For a Brownian particle that has to escape through *N*_*w*_ partially absorbing windows of size *a* located on a spherical surface, the escape time $\bar{\tau }$ is given by [57]

$$\begin{array}{c} \bar{\tau }=\frac{\left|\Omega \right|}{2\pi \kappa N_{w}a^{2}}, \end{array}$$

where |Ω| is the volume of the diffusing region, *κ* is partially absorbing constant that reflects the effect of the polymer on the dynamics of the moving particle. In the PSD, the accessible region Ω is the space occupied by the polymer.

Using the previous scaling laws (Fig 1B), we aim now at estimating how the number of escaping windows *N*_*w*_ depends on the random connectors *N*_*c*_. We start with the asymptotic behavior for the volume $|\Omega |∼R_{g}^{3}∼{\left(k_{rg}N_{c}^{-\nu }+r_{g}^{\infty }\right)}^{3}$, we next approximated the size of the escaping window *a* as the average pore size *ζ* (Fig 2D), $a∼\zeta ∼k_{\zeta }N_{c}^{-\gamma }+\zeta^{\infty }$. Then, the mean escape time can be rewritten as:

$$\begin{array}{c} \bar{\tau }=\frac{\left|\Omega \right|}{2\pi \kappa N_{w}a^{2}}∼\frac{{\left(k_{rg}N_{c}^{-\nu }+r_{g}^{\infty }\right)}^{3}}{{\left(k_{\zeta }N_{c}^{-\gamma }+\zeta^{\infty }\right)}^{2}N_{w}\left(N_{c}\right)}∼k_{\tau }N_{c}^{\eta }+\tau^{0}. \end{array}$$

Finally, we found:

$$\begin{array}{c} N_{w}\left(N_{c}\right)∼\frac{r_{g}^{\infty 3}}{\zeta^{\infty 2}k_{\tau }}[N_{c}^{-\eta }+\frac{3k_{rg}}{r_{g}^{\infty }}N_{c}^{-(\nu +\eta )}+\frac{2\gamma k_{\zeta }}{\zeta^{\infty }}N_{c}^{-(\eta +\gamma )}]. \end{array}$$ (8)

To conclude, the number of escaping windows is inversely proportional to the escaping time $∼N_{c}^{\eta }$.

### 2.7 Discussion and concluding remarks

**Fig 7:**
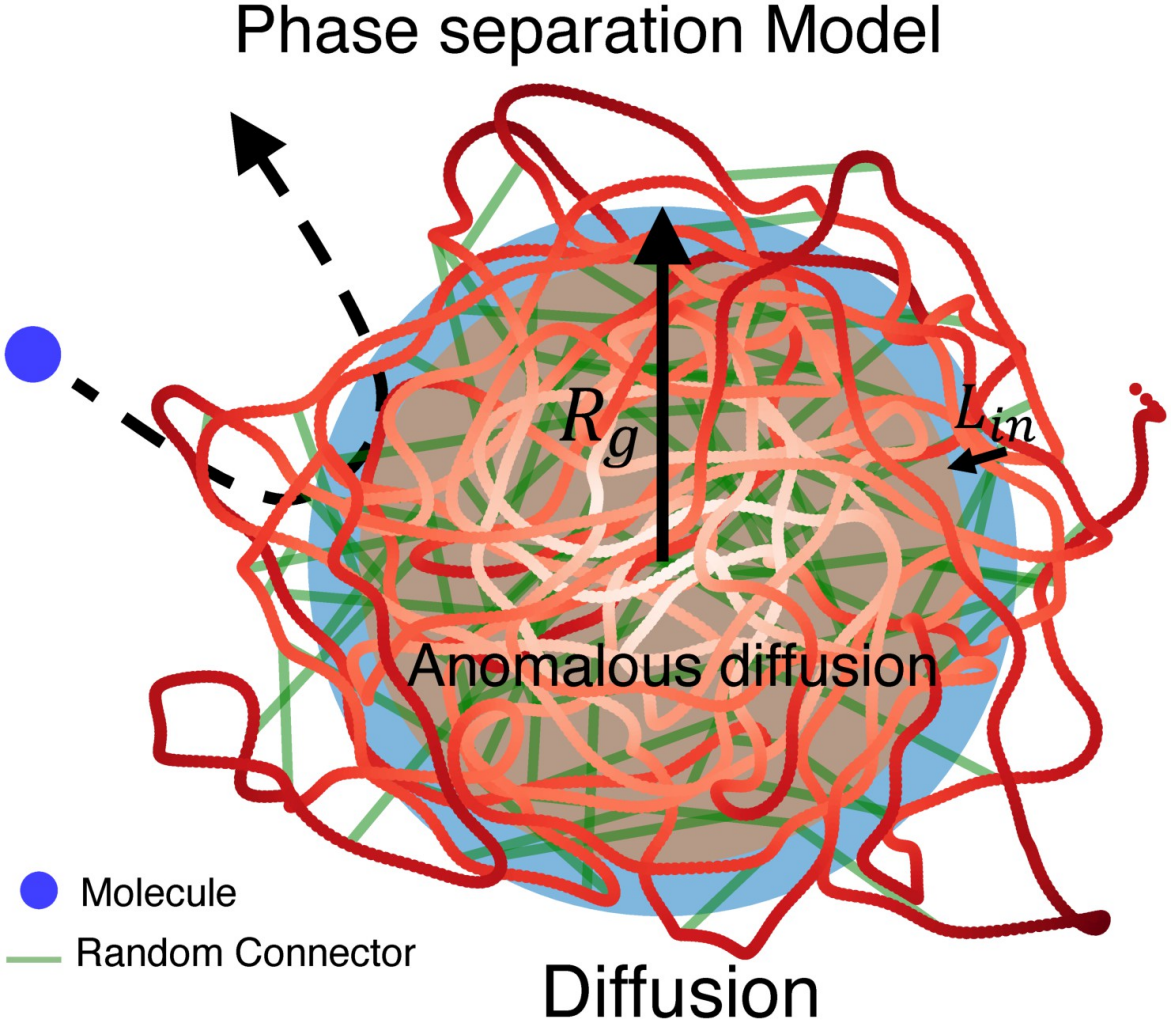
Summary of a Phase Separated Domain, described by a RCL-volume extrusion model, revealing a transition from anomalous diffusion to normal diffusion near the boundary.

We demonstrated here that the PSD can result from multiple connectors that would condense chromatin fiber (Fig 7). Using polymer model, scaling laws and numerical simulations, we found that a PSD can isolate diffusing molecules. Using a monomer resolution of 3kbp, corresponding to *σ* = 30nm, and *τ*_*MD*_ ≈ 0.02s (used in semi-dilute polymer solutions [43, 62]), the presence of *N*_*c*_ ∼ 50 leads to a PSD region of size 〈*R*_*g*_〉 ≃ 1.5 *μm*. In this context the resident time of a random particle is 〈*τ*_*in*_〉 ≃ 0.3s, while the escape time from the center of PSD is 〈*τ*_*e*_〉 ≃ 0.7s. Interestingly, these time scales are quite different from the life time of this PSD which depends on the dynamics of cross-linkers. We also reported here a boundary layer of 10–15% of the PSD size that can prevent stochastic particles from fully penetrating. Finally, we propose to use the mean escape time to quantify the ability of the PSD to retain particles inside and to measure the degree of isolation.

The present study suggests that adding connectors to a polymer model representing a flexible structure such as a TAD could lead to a region that shares the physical property of a PSD: membraneless, dynamical, and spatially organized assemblies of biomolecules interacting with a polymer that models a nucleic acid. Further more The present study shows that single particle trajectories (SPTs) can be used to analyze the properties of PSDs based on the distribution of their anomalous exponents. It would be interesting to estimate the PSD organization and the mean number of cross-linkers from the distribution of anomalous exponent, extracted from future SPT experiments. This reverse engineering problem can be addressed using the *α*-exponent curves from Fig 4I and 4J. Finally, the present polymer model approach suggests that PSD do not have a potential well signature to retain particles, as is the case for other nanodomains such as lipid raft (Calcium nanodomain at synapses or postsynaptic density in dendritic spines).

In the present manuscript, we did not account for histone local interactions such as histone-tail acetylation or nucleosomes attractive interactions with each other, which are made at a local distance. Acetylation or histone depletion could affect the chromatin dynamics in PSD, a subject that should be further explored. Indeed, we deployed here a coarse-grained model with a larger spatial resolution of few kbps larger, than nucleosome-nucleosome interactions.

Although the present cross-linker model contains a static realization of cross-linkers, it can be used to model PSD as defined above by weak, multivalent interactions among molecules such as proteins and a polymer. Indeed, the generalized (RCL) cross-linker polymer model accounts for chromatin chain fluctuations and thus boundary opening and closing. It is not clear what would be gained by adding dynamic and mobile crosslinkers and whether it would increase significantly chromatin chain fluctuations [58], compared to the polymer fluctuations already obtained by fixed cross-linkers. In addition, we reported here that the fluctuating chain dynamics results in transient opening and closing of windows that could allow diffusing molecules to be exchanged in a time scale of seconds [59] that we showed here control diffusing molecules in and out of the PSD domain. To conclude, the present model remains quite general as the residence time of 1–2 min for CFCF and 22 min for cohesin [60] is much longer than the time scale of few seconds of diffusing molecules. Thus the cross-linker model is applicable for studying transient events of few seconds such as three-dimensional TF diffusion, with a diffusion constant of few *μm*^2^/*s*, and a binding rate of 1*s*^−1^. It would take less than a second to bind and thus it would not be much affected by any additional fluctuations due to removal or addition of cross-linkers.

Loop extrusion phenomena was not explicitly accounted for here by our model, as it would require to model the extrusion process from cross-linkers. Here, we considered an effective model with static cross-linkers that does not require additional parameters to model their dynamics. Our model allows to investigate the dynamics of phase-separated domain at steady-state. Adding the loop extrusion mechanism would probably add minor modifications of the PSD, because it already contains tens of connectors, as suggested here: thus adding few loops at a time should not perturb the stable PSD, contrary to TAD morphology, that could be significantly reorganized.

Interestingly, fluorescence imaging revealed that inert molecules are expelled from the HP1 condensate in cells [26]. We recall that the concentration in the HP1 spots is much lower than that of in vitro HP1 droplets [61] so that some spots of high HP1 concentration in cells do not necessarily form droplet-like condensates in cells. However, this property of expelling inert molecules could be explained by our model (Fig 3A), where a small amount of cross-linkers (Fig 3I), leads to molecular trajectories with anomalous exponent > 1 (super-diffusion). This process results in expelling inert molecules. However, this effect disappears in a high condensed phase (N>600 connectors), where the motion remains sub-diffusive, associated with a higher degree of isolation.

Future analysis could focus on the formation of a PSD from an already existing TAD.

We proposed here that phase separated domains could result from adding connectors to TADs. This transformation shows the continuity for constructing PSD nanodomains from TADs, as a reversible process by simply modulating the number of cross-linkers (cohesion and CTCF). We thus predict that it could be possible to generate transitions between these two structures by simply adding or removing connectors, a process that could controlled by remodelers.

Finally, the present model of beads connected by spring could be generalized in a network of interacting scaffolding proteins present in neuronal synapses at the post-synaptic density [18–20]. The ensemble produces a phase separation domain that can regulate membrane receptors. However, we reported here that PSD generated by polymers do not generate a long-range attracting field: this is in contrast with synaptic nanodomains [19–21]. Probably the mechanism of phase separation in both cases is quite distinct: polymer constant reorganization can generate physical constrain, while molecular interactions at membrane induces an attractor by possibly deforming membranes. To conclude two and three dimensional polymer networks provide a mechanistic representation of phase separation that regulate local processes such as protein trafficking, transcription, plasticity and possibly many more.

## 3 Methods

The method is separated into three sections: we first present the characteristics of the cross-linked polymer models and the associated energy. Second, we summarize our simulation procedure. Third, we expand the computation associated to the scaling law for the mean escape time from a PSD.

### 3.1 Generalized random cross-linker polymer model to describe dense chromatin phases

#### 3.1.1 Construction of the polymer chain from potential well

We present here an extension of the random cross-linker model [40] that includes volume excluded interactions. This extension uses bead-spring polymer model, originating from the Kremer-Grest [42] coarse-grained model [43, 62, 63]. The model is constructed as follows: we consider a bead-spring polymer with a total of *N*_*mon*_ monomers where we have added *N*_*c*_ cross-linkers located at random positions. Each of *N*_*mon*_ interacting monomer of the polymer chain corresponds to 3 kbp, with size *σ* = 30*nm* and their dynamics is described by the potential energy which is the sum of several terms for the vector position of all beads $\left({\vec{r}}_{1},‥{\vec{r}}_{N}\right)$:

1. **The Lennard-Jones potential** $U_{LJ}({\vec{r}}_{1},‥{\vec{r}}_{N})$ describes the excluded volume interactions. We took for *U*_*LJ*_ a truncated and shifted Lennard-Jones potential: two beads repel when their distance is less than 2^1/6^*σ*, which corresponds to the minimum of the potential:
  $$\begin{array}{c} U_{LG}\left(r\right)=\{\begin{array}{cc} 4\epsilon [(\frac{\sigma }{r}{)}^{12}-(\frac{\sigma }{r}{)}^{6}+\frac{1}{4}] & r\leq r_{c}\\ 0 & r>r_{c}, \end{array} \end{array}$$ (9)
  where *r* is the distance between any two monomers while the cutoff distance *r*_*c*_ = 2^1/6^*σ* conserves only the repulsive contribution. The energy scale is *ϵ* = *κ*_*B*_*T*, where *T* = 300 K.
2. **Non-linear elastic potential (FENE)**. The linear connectivity of the chain is ensured by bonding nearest-neighbours monomers with the finitely extensible non-linear elastic potential (FENE): the energy $U_{FENE}({\vec{r}}_{1},‥{\vec{r}}_{N})$ is associated to the backbone of the polymer chain. This potential enforces the connectivity of the chain, so that two consecutive particles cannot be distant by more than *R*_0_ = 1.5*σ*.
  $$\begin{array}{c} U_{FENE}\left(r\right)=\{\begin{array}{cc} -0.5\kappa R_{0}^{2}\text{ln}(1-(r/R_{0}{)}^{2}) & r\leq r_{c}\\ \infty & r>r_{c}, \end{array} \end{array}$$ (10)
  where *κ* = 30*ϵ*/*σ*^2^ is the spring constant and *R*_0_ = 1.5*σ* is the maximum extension of the elastic FENE bond.
3. **The bending energy** *U*_*bend*_. The stiffness of the polymer is quantified by the bending energy which depends on the cosine of the angle between two consecutive bonds along the chain. The bending energy $U_{bend}({\vec{r}}_{1},‥{\vec{r}}_{N})$ penalizes consecutive bond vectors ${\vec{b}}_{i}={\vec{r}}_{i+1}-{\vec{r}}_{i}$ that are not parallel. Using the monomers positions ${\vec{r}}_{i}$ along the chain, the analytical expression is given by
  $$\begin{array}{c} U_{\text{bend}}\left({\vec{r}}_{i-1},{\vec{r}}_{i},{\vec{r}}_{i+1}\right)=\kappa_{\theta }(1-\frac{\left({\vec{r}}_{i+1}-{\vec{r}}_{i}\right)\cdot \left({\vec{r}}_{i}-{\vec{r}}_{i-1}\right)}{|{\vec{r}}_{i+1}-{\vec{r}}_{i}\left|\;\right|{\vec{r}}_{i}-{\vec{r}}_{i-1}|}), \end{array}$$ (11)
  where *κ*_*θ*_ = 5*κ*_*B*_*T* is the bending constant as the Kuhn’s length of the 30-nm fiber is *l*_*K*_ = 300 nm, parameters obtained from [43].
4. **Harmonic potential** *U*_*harm*_ **between random connectors**. The presence of loops is implemented with an harmonic potential to add *N*_*c*_ cross-linkers between randomly chosen monomers. The energy is given by
  $$\begin{array}{c} U_{harm}\left(r_{i,j}\right)=\frac{k_{rc}}{2}r_{i,j}^{2}, \end{array}$$ (12)
  where *k*_*rc*_ = 0.5*σ*^2^/*ϵ* is the spring constant, $r_{i,j}=|{\vec{r}}_{i}-{\vec{r}}_{j}|$ the distance between two non-nearest-neighbours monomers connected by a random connector.

To summarize the polymer chain is described by the following energy term:

$$\begin{array}{c} H_{INT}\left(r\right)=\sum_{i,j}^{N_{mon}}U_{LG}\left({\vec{r}}_{1},‥{\vec{r}}_{N}\right)+\sum_{i=1}^{N_{mon}-1}U_{FENE}\left(r_{i,i+1}\right)+\sum_{i=2}^{N_{mon}-1}U_{\text{bend}}\left({\vec{r}}_{i-1},{\vec{r}}_{i},{\vec{r}}_{i+1}\right)+\sum_{k=(k_{i},k_{j})}^{N_{c}}U_{harm}\left(r_{k_{i},k_{j}}\right). \end{array}$$ (13)

#### 3.1.2 Langevin’s dynamics of the polymer chain

The dynamics of the chain is described by the Langevin equation:

$$\begin{array}{c} m\frac{dv}{dt}=-m\gamma v-\nabla H_{INT}+\sqrt{2dD}\dot{\eta }. \end{array}$$ (14)

where *η* is zero-mean Gaussian noise. We recall that *N*_*mol*_ molecules and *N*_*mon*_ monomers of size *σ* diffuse with diffusion coefficient $D=\frac{\kappa_{B}T}{\gamma }$. The molecule-molecule and molecule-monomer interactions are defined according the truncated Lennard-Jones potential 9. Along the chain, we positioned *N*_*bs*_ binding sites on monomers: a free molecule can then be attached to a binding site when their relative distance is *d* < 2 ⋅ 2^1/6^*σ* via a Lennard-Jones attractive potential with *ϵ* = 5*κ*_*B*_*T*. A molecule can attach to only one binding site, while each binding site can accommodate more than one binding molecule.

#### 3.1.3 Numerical implementation

The model has been investigated performing fixed-volume and constant-temperature Molecular Dynamics (MD) simulations with implicit solvent. The equations of motion are integrated using a velocity Verlet algorithm and Langevin thermostat with temperature *T* = *κ*_*B*_ and damping constant $\gamma =0.5\tau_{MD}^{-1}$ where *τ*_*MD*_ = *σ*(*m*/*ϵ*)^1/2^ is the Lennard-Jones time scale. In the case of semi-dilute polymer solutions, it is equivalent to *τ*_*MD*_ ≈ 0.02 s [43].

The integration time step is set to Δ*t* = 5 ⋅ 10^−3^*τ*_*MD*_. The length of each MD run for the system composed by an already equilibrated RCL-polymer and the diffusive particles is equal to 5 ⋅ 10^6^ simulation steps (2.5 ⋅ 10^4^*τ*_*MD*_) after an equilibrium run of 10^6^ simulation steps. The effect of random cross-linking is obtained by considering 10^2^ different random polymer connectivities.

### 3.2 Scaling law for the mean escape time from a PSD

The escape time $\bar{\tau }$ for a Brownian particle escaping through *N*_*w*_ partially absorbing windows of size *a* located on a spherical surface, is given by [57]:

$$\begin{array}{c} \bar{\tau }=\frac{\left|\Omega \right|}{2\pi \kappa N_{w}a^{2}}, \end{array}$$

where |Ω| is the volume of the diffusing region, *κ* is partially absorbing constant that reflects the effect of the polymer on the dynamics of the moving particle. In the PSD, the accessible region Ω is the space occupied by the polymer. We show here the detailed computations for estimating how the number of escaping windows *N*_*w*_ depends on the random connectors *N*_*c*_. We start with the asymptotic behavior for the volume $|\Omega |∼R_{g}^{3}∼{\left(k_{rg}N_{c}^{-\nu }+r_{g}^{\infty }\right)}^{3}$, we next approximated the size of the escaping window *a* as the average pore size *ζ*, $a∼\zeta ∼k_{\zeta }N_{c}^{-\gamma }+\zeta^{\infty }$. Then, we the mean escape time can be rewritten as:

$$\begin{array}{c} \bar{\tau }=\frac{\left|\Omega \right|}{2\pi \kappa N_{w}a^{2}}∼\frac{{\left(k_{rg}N_{c}^{-\nu }+r_{g}^{\infty }\right)}^{3}}{{\left(k_{\zeta }N_{c}^{-\gamma }+\zeta^{\infty }\right)}^{2}N_{w}\left(N_{c}\right)}∼k_{\tau }N_{c}^{\eta }+\tau^{0}. \end{array}$$

We can the isolate the espression for the number of escaping windows as a funtion of the number of connectors *N*_*c*_:

$$\begin{array}{c} N_{w}\left(N_{c}\right)∼\frac{k_{rg}^{3}N_{c}^{-3\nu }+3k_{rg}^{2}N_{c}^{-2\nu }r_{g}^{\infty }+3k_{rg}N_{c}^{-\nu }r_{g}^{\infty 2}+r_{g}^{\infty 3}}{\left(k_{\zeta }^{2}N_{c}^{-2\gamma }+\zeta^{\infty 2}+2k_{\zeta }N_{c}^{-\gamma }\zeta^{\infty })(k_{\tau }N_{c}^{\eta }+\tau^{\infty }\right)} \end{array}$$

that can be rearranged as

$$\begin{array}{c} N_{w}\left(N_{c}\right)∼\frac{r_{g}^{\infty 3}}{\zeta^{\infty 2}\tau^{\infty }}[\frac{1+\frac{3k_{rg}}{r_{g}^{\infty }}N_{c}^{-\nu }+O(N_{c}^{-2\nu })}{\left(1+\frac{2k_{\zeta }}{\zeta^{\infty }}N_{c}^{-\gamma }+O(N_{c}^{-2\gamma }))(1+\frac{k_{\tau }}{\tau^{\infty }}N_{c}^{\eta }\right)}]. \end{array}$$

In the limit *N*_*c*_ large we can set

$$\begin{array}{c} 1+\frac{k_{\tau }}{\tau^{\infty }}N_{c}^{\eta }∼\frac{k_{\tau }}{\tau^{\infty }}N_{c}^{\eta } \end{array}$$

and get

$$\begin{array}{c} N_{w}\left(N_{c}\right)∼\frac{r_{g}^{\infty 3}}{\zeta^{\infty 2}k_{\tau }}[\frac{N_{c}^{-\eta }+\frac{3k_{rg}}{r_{g}^{\infty }}N_{c}^{-(\nu +\eta )}+O(N_{c}^{-2\nu })}{1+\frac{2k_{\zeta }}{\zeta^{\infty }}N_{c}^{-\gamma }+O(N_{c}^{-2\gamma })}]. \end{array}$$

Expanding the denominator we then get

$$\begin{array}{c} N_{w}\left(N_{c}\right)∼\frac{r_{g}^{\infty 3}}{\zeta^{\infty 2}k_{\tau }}(N_{c}^{-\eta }+\frac{3k_{rg}}{r_{g}^{\infty }}N_{c}^{-(\nu +\eta )}+O(N_{c}^{-2\nu }))[1+(\frac{2\gamma k_{\zeta }}{\zeta^{\infty }}N_{c}^{-\gamma }+O(N_{c}^{-2\gamma }))]. \end{array}$$

Finally, we get

$$\begin{array}{c} N_{w}\left(N_{c}\right)∼\frac{r_{g}^{\infty 3}}{\zeta^{\infty 2}k_{\tau }}(N_{c}^{-\eta }+\frac{3k_{rg}}{r_{g}^{\infty }}N_{c}^{-(\nu +\eta )}+\frac{2\gamma k_{\zeta }}{\zeta^{\infty }}N_{c}^{-(\eta +\gamma )}). \end{array}$$

## Supporting information

S1 Fig Radial distribution and pair correlation functions. **radial distribution function**. First column: molecules radial distribution function *g*_mol_(*r*) for different density; Second column: polymer radial distribution function *g*_mon_(*r*). Third column: molecules-monomers pair correlation function *g*_mol_(*r*). Fourth column: molecule-molecule pair correlation function *g*_mol,mol_(*r*). Fifth column: monomer-monomer pair correlation function *g*_mon,mon_(*r*).
(PDF)
