# Supplementary material for: Chromatin phase separated nanoregions explored by polymer cross-linker models and reconstructed from single particle trajectories
Source: PLoS Comput Biol. 2024 Jan 24;20(1):e1011794. doi: 10.1371/journal.pcbi.1011794 (PMC10843633; doi:10.1371/journal.pcbi.1011794)
Supplement: S1 Fig — radial distribution function. First column: molecules radial distribution function gmol(r) for different density; Second column: polymer radial distribution function gmon(r). Third column: molecules-monomers pair correlation function gmol(r). Fourth column: molecule-molecule pair correlation function gmol,mol(r). Fifth column: monomer-monomer pair correlation function gmon,mon(r). (PDF) [file pcbi.1011794.s001.pdf]

Chromatin phase separated nanoregions regulated by cross-linkers and explored by single particle trajectories: Supplementary Material

A, Papale[1], D. Holcman[1,2]
[1] *Group of Computational Biology and Applied Mathemathics, Ecole Normale Supérieure, IBENS, Université PSL, 75005 Paris, France and*
[2] *Churchill College, University of Cambridge, CB30DS, United Kingdom*
(Dated: March 25, 2023)

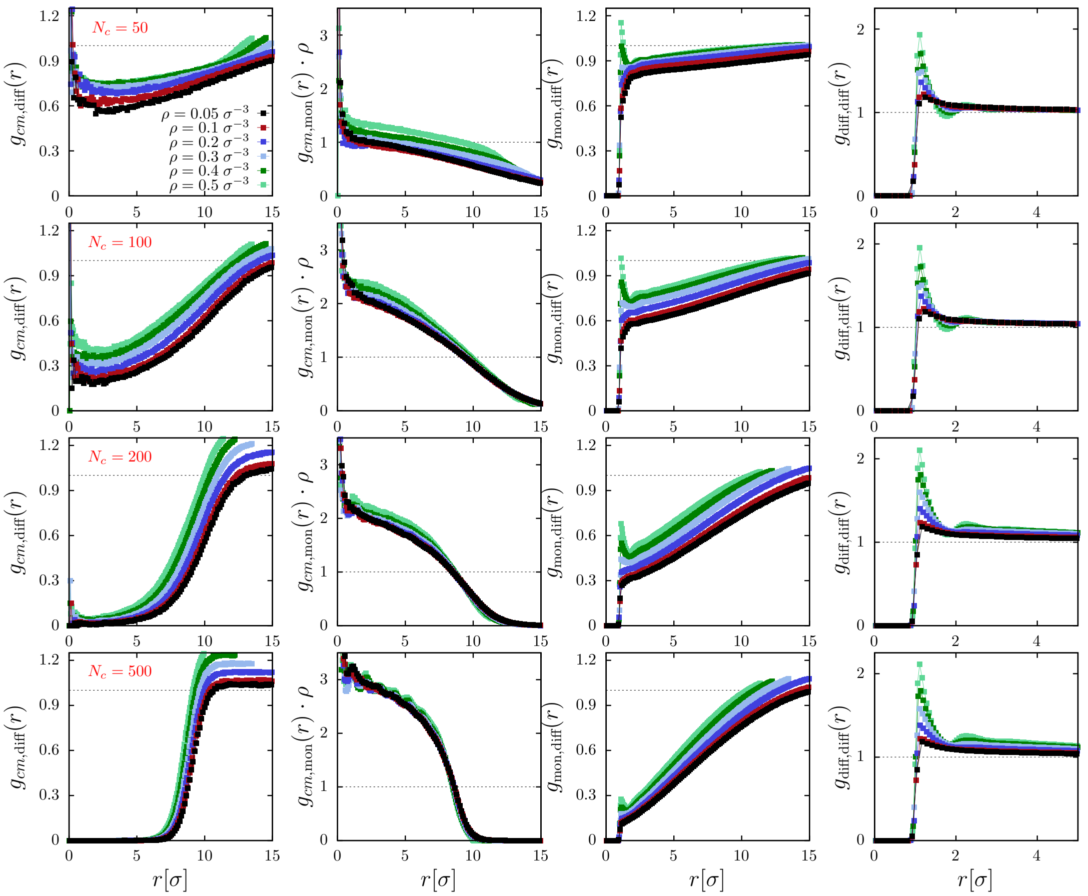

FIG. S1. First column: molecules radial distribution function $g_{\mathrm{mol}}(r)$ for different density; Second column: polymer radial distribution function $g_{\mathrm{mon}}(r)$. Third column: molecules-monomers pair correlation function $g_{\mathrm{mol}}(r)$. Fourth column: molecule-molecule pair correlation function $g_{\mathrm{mol,mol}}(r)$. Fifth column: monomer-monomer pair correlation function $g_{\mathrm{mon,mon}}(r)$.
